# Androgen levels in autism spectrum disorders: a systematic review and meta-analysis

**DOI:** 10.3389/fendo.2024.1371148

**Published:** 2024-05-08

**Authors:** Zhao Wang, Bohan Zhang, Chenyu Mu, Dan Qiao, Huan Chen, Yan Zhao, Huixian Cui, Rong Zhang, Sha Li

**Affiliations:** ^1^ Department of Anatomy, Neuroscience Research Center, Hebei Medical University, Shijiazhuang, China; ^2^ Hebei Key Laboratory of Neurodegenerative Disease Mechanism, Shijiazhuang, China; ^3^ School of Nursing, Hebei Medical University, Shijiazhuang, China; ^4^ Neuroscience Research Institute, Key Laboratory for Neuroscience, Ministry of Education of China, Peking University, Beijing, China; ^5^ Key Laboratory for Neuroscience, National Committee of Health, Department of Neurobiology, School of Basic Medical Sciences, Peking University, Beijing, China; ^6^ Autism Research Center of Peking University Health Science Center, Beijing, China; ^7^ The Key Laboratory of Neural and Vascular Biology, Ministry of Education, Shijiazhuang, China

**Keywords:** autism spectrum disorder, androgen, sex differences, hormone, meta-analysis

## Abstract

**Background:**

Accumulating evidence suggests that the autism spectrum disorder (ASD) population exhibits altered hormone levels, including androgens. However, studies on the regulation of androgens, such as testosterone and dehydroepiandrosterone (DHEA), in relation to sex differences in individuals with ASD are limited and inconsistent. We conducted the systematic review with meta-analysis to quantitatively summarise the blood, urine, or saliva androgen data between individuals with ASD and controls.

**Methods:**

A systematic search was conducted for eligible studies published before 16 January 2023 in six international and two Chinese databases. We computed summary statistics with a random-effects model. Publication bias was assessed using funnel plots and heterogeneity using I^2^ statistics. Subgroup analysis was performed by age, sex, sample source, and measurement method to explain the heterogeneity.

**Results:**

17 case-control studies (individuals with ASD, 825; controls, 669) were assessed. Androgen levels were significantly higher in individuals with ASD than that in controls (SMD: 0.27, 95% CI: 0.06–0.48, *P*=0.01). Subgroup analysis showed significantly elevated levels of urinary total testosterone, urinary DHEA, and free testosterone in individuals with ASD. DHEA level was also significantly elevated in males with ASD.

**Conclusion:**

Androgen levels, especially free testosterone, may be elevated in individuals with ASD and DHEA levels may be specifically elevated in males.

## Introduction

Autism spectrum disorder (ASD) is a neurodevelopmental disorder characterised by impaired social communication, limited interests, and repetitive behaviours. Although the aetiology of ASD is not fully clarified, both genetic and environmental factors reportedly play important roles. In the United States, the prevalence of ASD among children with 8 years old has reached 1/36, suggesting that ASD has been one of the most common neurodevelopmental psychiatric disorders (1). Gender differences exist in the incidence of ASD, with a male to female ratio of about 4~6:1 (2). The impact of sex hormones on brain development may be an important factor contributing to gender differences in ASD. Androgens are key regulators of male sexual differentiation and the development of normal male phenotypes. Testosterone, the major human androgen, plays a dominant role in sexual dimorphism (3). Genetic and environmental effects modulate the gene expression of steroid-metabolising enzymes in the steroidogenic cascade, consistent with the expression regulation of the corresponding receptors, implying a complex mechanism of androgen action. Thus, early and increasing awareness of the androgen levels among individuals with ASD is a crucial component for the early diagnosis of ASD. Despite extensive studies on ASD and androgens, we found that androgen changes were reported inconsistently in many individuals diagnosed with ASD.

Studies have shown abnormalities in steroid metabolism in individuals with ASD; however, the data are inconsistent because some studies have documented androgen excess in children and adults with ASD (4, 5), Geier and Geier (4) conducted a study where they demonstrated significantly elevated levels of serum testosterone, free testosterone (FT), dehydroepiandrosterone (DHEA), and androstenedione in individuals with ASD. These findings suggest a possible link between androgen excess and ASD, which could explain some of the behavioural and cognitive characteristics observed in individuals with this condition. Contrary to these findings, other studies have reported androgen deficiency in individuals with ASD (6, 7). For instance, a study by Croonenberghs et al. (6) found that the significant decrease in serum testosterone concentration in male youngsters with autism suggest that the turnover of testosterone may take part in the pathophysiology of autism. Still, other studies have found no significant difference in androgen levels between individuals with ASD and controls. For instance, a study by Tordjman et al. (8) found no significant difference in the serum testosterone levels in prepubertal or adolescent boys with ASD and age-matched control individuals. To further complicate the picture, a study on amniotic fluid by Auyeung et al. (9) found altered hormone levels, including higher androgen levels, in children with ASD. This finding suggests that steroid metabolism abnormalities may already be present during prenatal development.

So far, there are very few systematic review and meta-analysis of case-control studies which have specifically evaluated the androgen levels in individuals with ASD. There is only one meta-analyses of literature related to the effect of prenatal high androgen exposure on autism, but the androgen theory of autism is neither confirmed nor refuted by the existing association studies in that review (10). We aimed to determine the differences in androgen levels between individuals with ASD and controls to provide the basis for the androgen theory of autism. Meanwhile, we further investigated the effect of factors, such as sex, age, measurement method, and sample source, on the androgen levels in individuals with ASD.

## Materials and methods

### Literature search

A systematic literature search was performed on six international databases (PubMed, Cochrane Library, Web of Science, Sinomed, Embase, and OVID) and two Chinese databases (Wanfang Data and CNKI), and eligible literature on ASD and androgens was included. The search period spanned from the database inception to 26 January 2023. We checked the references of related literature to further identify research on the study topic. The search terms included combinations of medical subject title terms and free words. Chinese search terms included ‘autism’, ‘autism spectrum disorder’, ‘autistic’, ‘autistic disorder’, ‘Asperger’s syndrome’, ‘androgen’, ‘testosterone’, and ‘dehydroepiandrosterone’. The English search terms included ‘autism,’ ‘infantile autism’, ‘autism spectrum disorder’, ‘Asperger’s syndrome’, ‘Kanner syndrome’, ‘Androstenedione’, ‘4-Androstene-3,17-dione’, ‘5 alpha-Dihydrotestosterone’, ‘Epitestosterone’, ‘androgen’, ‘testosterone’, and ‘dehydroepiandrosterone’. Our literature search on PubMed, as an example, is shown in **Supplementary Table S5**. Our search strategy followed the PRISMA guidelines (11).

### Eligibility criteria

**Figure 1:**
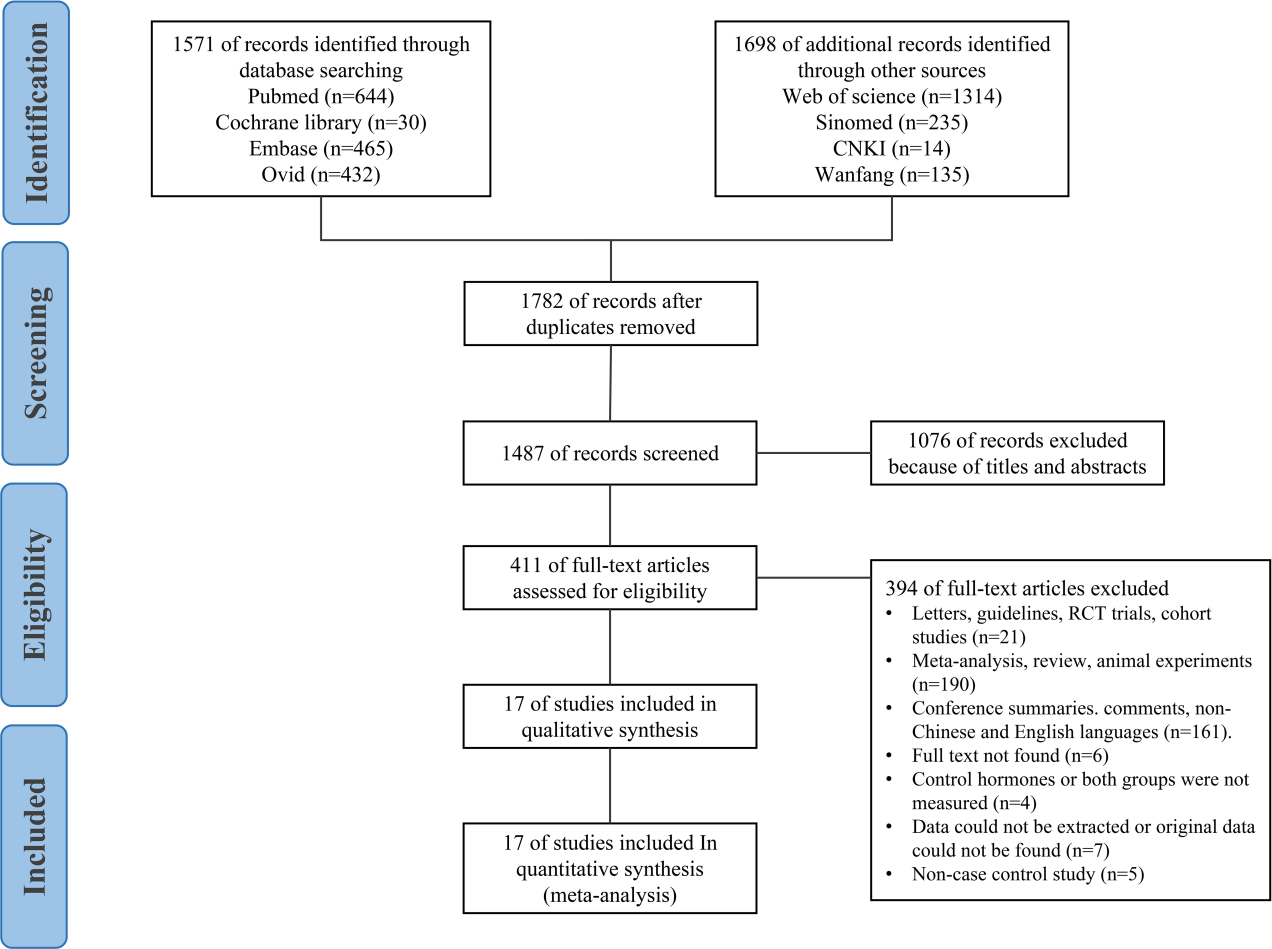
Flowchart for searching and selection of the included studies.

The inclusion criteria for studies in this systematic review were as follows: 1) case-control studies; 2) studies including individuals with ASD according to standard operationalised diagnostic criteria (e.g. Diagnostic and Statistical Manual of Mental Disorders, 5th edition [DSM-V] or 4th edition [DSM-IV], International Classification of Diseases, 10th Revision [ICD-10]); 3) studies including participants from population or clinical samples of individuals, including those diagnosed with medical conditions affect androgen levels; 4) studies that reported unique data on androgen or androgen-related metabolites. 5) for repeated publications, only those with the most detailed description of research methods, complete data, and well-controlled confounding factors were accumulated;

Our exclusion criteria were as follows: 1) non-case-control studies; 2) studies in which the inclusion criteria do not meet the diagnostic criteria for ASD; 3) studies with no hormone level measurement, no available data, data that could not be extracted, or incomplete data and only abstracts without full text; 4) reviews, case reports, case analyses, editorials, animal studies, letters, conference abstracts, randomised controlled trials with experiments, or cohort studies; 5) studies involving measurement of the foetal hormone levels in mothers with ASD during pregnancy. The study selection process is shown in **Figure 1**.

### Data extraction

The titles and abstracts of studies identified in the initial search were imported into EndNote for preliminary screening. Literature screening and data extraction were completed independently by two researchers, and inconsistent results were arbitrated by a third reviewer. The following content was extracted: 1) General information: literature title, first author, publication year, confounding factor matching, sample source, and detection method; 2) Participants: sample size, age, and sex ratio; 3) Case-control study method: definition of cases, representativeness of cases, selection of controls, definition of controls, determination of exposure, and non-response rate; 4) Androgen level measurement type (e.g. total testosterone [TT]); 5) Research results: Androgen levels in individuals with ASD and control groups. The combined results are expressed as means ± standard deviations. If only median and interquartile range data were provided, they were converted to mean and standard deviation; mean and standard error data were also converted to mean and standard deviation (12, 13). The study characteristics are shown in **Supplementary Table S1** and **Supplementary Table S2**.

### Review protocol

The detailed protocol is available at PROSPERO (identifier CRD42022325221).

### Methodological quality evaluation of the included studies

Two reviewers independently assessed the quality of the included studies using the Newcastle–Ottawa Scale (NOS) (14) for case-control studies. The scores were assigned on a 9-point scale, based on three aspects: study population selection, comparability, and outcome. Except for comparability, which was scored with 2 points, each criterion was given 1 point, where higher scores indicated better quality. In this study, 1–3 points, 4–6 points, and 7–9 points were the scores for low-, medium-, and high-quality literature, respectively.

### Statistical analysis

Data analyses were performed using Reviewer Manager 5.3 (Copenhagen: Nordic Cochrane Centre, The Cochrane Collaboration, 2014) and STATA/MP 14.0 (Stata Corp., College Station, TX, USA). The collected data were considered as continuous variables, and when levels of the same hormones were measured using different units in different studies, we used the standardised mean difference (SMD) to determine pooled effects. We used a random-effects model with generic inverse variance to pool the effects and their corresponding 95% confidence intervals (CIs). The I^2^ statistic was used to test for heterogeneity of the included studies (15, 16), whereby I^2^ >50% indicates substantial heterogeneity (15). When the heterogeneity test result (I^2^) of the included studies was <50%, the fixed-effects model was used for the meta-analysis; alternatively, when the heterogeneity test result of the included studies was >50%, the random-effects model was used for the meta-analysis (17). Thereafter, the sources of heterogeneity were investigated through sensitivity tests, subgroup analyses, and meta-regression analyses. When there were more than five included studies, a funnel plot was used to analyse potential publication bias. Sensitivity analysis was used to test for the stability of the meta-analysis results.

## Results

### Study identification and selection

From the initial literature search, 3,269 articles were retrieved (Ovid: 432 articles, PubMed: 644 articles, Sinomed: 235 articles, Web of Science: 1,314 articles, Cochrane Library: 30 articles, Embase: 465 articles, CNKI: 14 articles, Wanfang: 135 articles). We excluded 1,782 articles because of duplication. Furthermore, 1,076 studies were screened by titles and abstracts and excluded. Upon application of the eligibility criteria, 394 studies were further excluded. A total of 23 full-text articles were scrutinised, of which 17 case-control studies were eligible for inclusion in this review, including one article in Chinese (18) and 16 articles in English (5–8, 19–30).

### Characteristics of the studies

**Supplementary Table S1** presents the detailed characteristics of each study. The androgen levels are shown in **Supplementary Table S2**. The total number of participants in all 17 included studies was 1,494, including 825 (87.8% male and 12.2% female) individuals with ASD and 669 (83.4% male and 16.6% female) individuals in the control groups. In most of the studies, the individuals with ASD were diagnosed according to the DSM-IV criteria, but in four studies (18, 20, 22, 25), the DSM-V criteria were used. One study (8) used the DSM-III-R criteria to establish the diagnosis. Some studies used standardised diagnostic tools, including the ASD Diagnostic Observation Program (30) and the Childhood Autism Rating Scale (24).

Individuals with ASD were matched with control groups in terms of age and sex in all studies. Five of these studies examined the effect of body mass index (BMI) (5, 6, 19, 22, 23). Additionally, 10 studies reported blood androgen levels, two studies reported urinary androgen levels, and four studies reported salivary androgen levels. Only one study measured the androgen levels in both blood and saliva (20).

### Methodological quality evaluation of the included studies

The 17 studies included were published during 1995–2021. All the included studies were scored using the NOS scoring system. Seven, seven, and three studies received 9, 8, and 7 points, respectively. The general quality of the articles was high (**Supplementary Table S3**).

### Primary outcome

**Figure 2:**
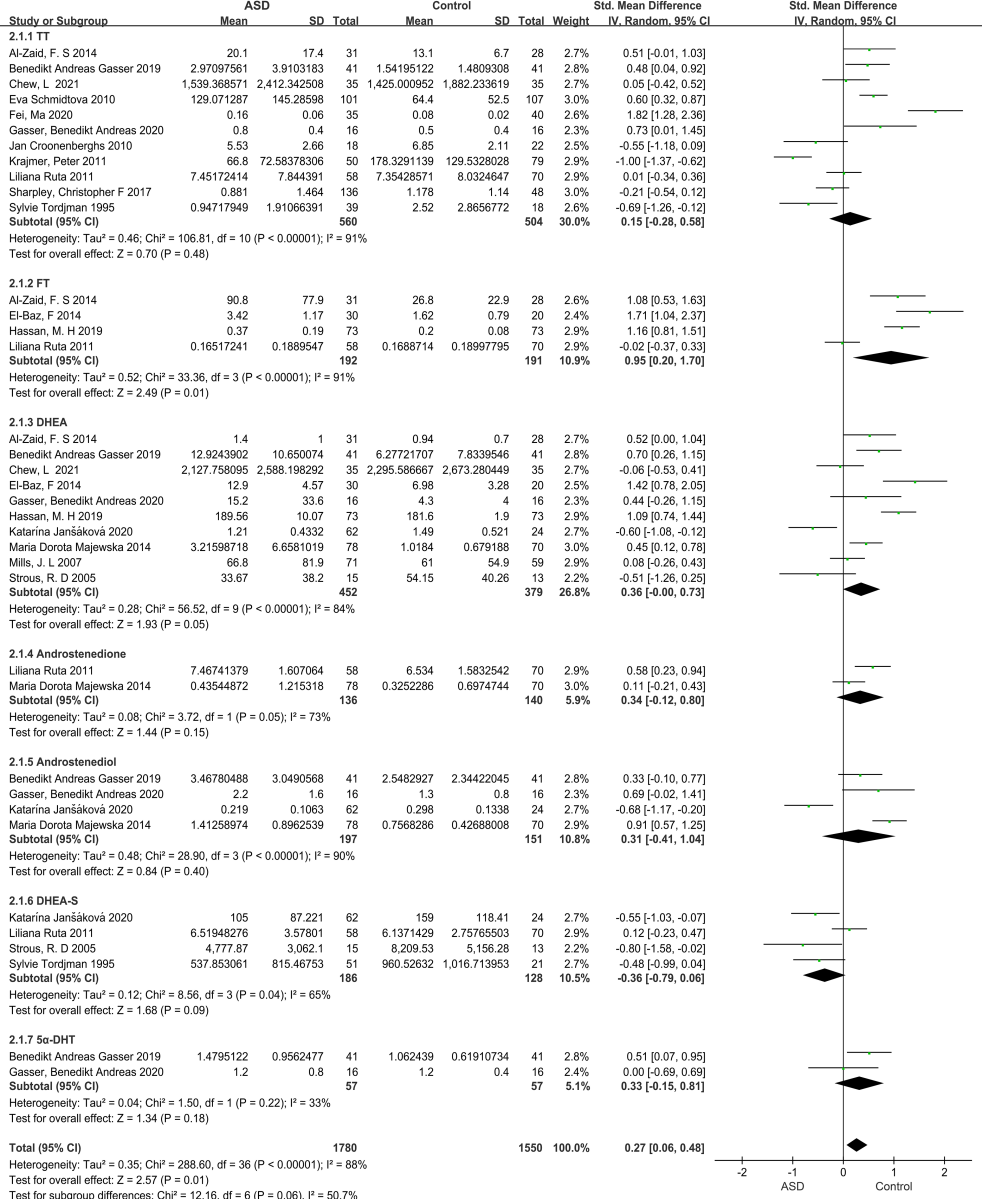
Forest plot of 17 studies comparing overall androgen levels between individuals with ASD versus Controls. The X-axis is the effect size of each study (usually a weighted average). For line segments parallel to the X-axis, a segment represents a study, and both ends of the segment are the upper and lower limits of the 95% confidence interval. In the line segment, the midpoint of the square is the effect value, and the size represents the weight of the research. The lines perpendicular to the X-axis are invalid lines, dividing the graph in two, with the left representing the experimental group and the right representing the control group. The diagonal intersection points of the black diamond block represent the combined effect size, the left and right tips represent the upper and lower limits of the 95% confidence interval after the merger, and the area represents the total sample size after the merger. SMD, Standardized mean difference; CI, confidence interval; ASD, Autism spectrum disorder; DHEA-s, Dehydroepitestosterone sulfate; 5a-DHEA, 5a-Dihydrotestosteron; FT, Free testosterone; TT, Total testosterone; DHEA, Dehydroepiandrosterone.

Our meta-analysis focused on androgen levels not only in individuals with ASD but also controls, based on data presented in 17 studies, providing 37 data points. The blood, urine, or saliva androgen concentrations (including TT, FT, DHEA, DHEA-S, androstenedione, androstenediol, 5α-dihydrotestosterone) in individuals with ASD to those in control groups were compared, and the combined effect size was significant (SMD: 0.27, 95% CI: 0.06–0.48, *P*=0.01), suggesting that the androgens levels in individuals with ASD were significantly higher than those in the control groups (**Figure 2**; **Table 1**). Four of the 37 SMD estimates were calculated for FT, the combined heterogeneity test was significant (X^2^ = 33.36, I^2^ = 91%, *P*<0.00001), and the combined effect size was significant (SMD: 0.95, 95% CI: 0.20–1.70, *P*=0.01). The results suggest that the FT level in the ASD group was significantly higher than that in the control group. The specific results for other hormones, namely, TT, DHEA-S, androstenedione, androstenediol, 5α-dihydrotestosterone, are shown in **Figure 2**.

**Table 1:** Main analysis and subgroup analyses of androgen.

Analysis	Number of studies	Meta-analysis			Heterogeneity analysis		
		SMD	95%CI	P	I^2^	X^2^	P
Main analysis							
Androgen	17	0.27	0.06 to 0.48	**0.01**	88%	288.60	<0.00001
Total testosterone	11	0.15	−0.28 to 0.58	0.48	91%	106.81	<0.00001
Free testosterone	4	0.95	0.20 to 1.70	**0.01**	91%	33.36	<0.00001
DHEA	10	0.36	−0.00 to 0.73	0.05	84%	56.52	<0.00001
Androstenedione	2	0.34	−0.12 to 0.80	0.15	73%	3.72	0.05
Androstenediol	4	0.31	−0.41 to 1.04	0.40	90%	28.90	<0.00001
DHEA-S	4	−0.36	−0.79 to 0.06	0.09	65%	8.56	0.09
5α-DHT	2	0.33	−0.15 to 0.81	0.18	33%	1.50	0.22
Secondary analysis							
TT							
Sex (males)	9	−0.07	−0.46 to 0.33	0.75	87%	63.48	<0.00001
Sex (females)	2	0.42	−0.07 to 0.90	0.09	20%	1.25	0.26
Age (prepubertal)	5	0.32	−0.52 to 1.15	0.46	93%	56.38	<0.00001
Age (postpubertal)	8	−0.13	−0.51 to 0.26	0.52	79%	34.04	<0.0001
Biological material (blood)	6	0.21	−0.48 to 0.90	0.56	90%	51.29	<0.00001
Biological material (salivary)	4	−0.00	−0.77 to 0.77	0.99	94%	50.25	<0.00001
Biological material (urine)	2	0.55	0.17 to 0.92	**0.004**	0%	0.34	0.56
Prepubescent male	4	−0.05	−0.84 to 0.73	0.90	90%	29.51	<0.00001
Postpubescent male	7	−0.22	−0.62 to 0.17	0.26	79%	28.44	<0.00001
Measurement methods (RIA)	2	0.57	−1.89 to 3.03	0.65	97%	38.77	<0.00001
Measurement methods (ELISA)	4	−0.03	−0.77 to 0.71	0.94	94%	50.2	<0.00001
Measurement methods (GC-MS/MS)	2	0.55	0.17 to 0.92	**0.004**	0%	0.34	0.56
Measurement methods (Others)	3	−0.08	−0.39 to 0.22	0.59	24%	2.63	0.27
DHEA							
Sex (males)	8	0.43	0.02 to 0.84	**0.04**	86%	50.18	<0.00001
Sex (females)	2	0.36	−0.04 to 0.75	0.08	0%	0.09	<0.00001
Age (prepubertal)	6	0.48	−0.03 to 0.99	0.07	89%	44.96	<0.00001
Age (postpubertal)	4	0.16	−0.33 to 0.66	0.52	64%	8.27	0.004
Biological material (blood)	7	0.40	−0.07 to 0.87	0.10	87%	47.88	<0.00001
Biological material (salivary)	2	−0.05	−1.12 to 1.01	0.92	88%	8.19	0.004
Biological material (urine)	2	0.59	0.18 to 1.01	**0.005**	0%	0.27	0.60
Prepubescent male	6	0.48	−0.03 to 0.99	0.07	89%	44.96	<0.00001
Postpubescent male	2	0.30	−0.43 to 1.02	0.42	77%	4.30	0.04
Measurement methods (RIA)	1	−0.51	−1.26 to 0.25	0.19	–	–	–
Measurement methods (ELISA)	2	1.17	0.86 to 1.47	**<0.00001**	0%	0.78	0.38
Measurement methods (GC-MS/MS)	4	0.23	−0.33 to 0.80	0.42	82%	16.5	0.0009

TT, total testosterone; FT, free testosterone; DHEA, dehydroepiandrosterone; DHEA-s, dehydroepiandrosterone sulfate; 5α-DHT, 5α-Dihydrotestosterone. The meaning of the bold values is that the differences are statistically significant (P < 0.05)

### Results of subgroup analysis

#### Subgroup analysis by sample source

Three sample sources (blood, urine, saliva) were compared for TT and DHEA levels. For the urine TT group, the heterogeneity test result was not significant (X^2^ = 0.34, I^2^ = 0%, *P*=0.56), but the combined effect size was significant (SMD: 0.55, 95% CI: 0.17–0.92, *P*=0.004). In contrast, the heterogeneity of the blood and saliva TT groups was significant, and the combined effect size was not significant (**Supplementary Figure S1**). Furthermore, the heterogeneity test result for the urine DHEA group was not significant (X^2^ = 0.27, I^2^ = 0%, *P*=0.60), while the combined effect size was significant (SMD: 0.59, 95% CI: 0.18–1.01, *P*=0.005). However, the heterogeneity of the blood and saliva DHEA groups was significant, and the pooled effect size was not significant (**Supplementary Figure S2**).

#### Subgroup analysis by sex

The heterogeneity test result for TT levels in males was significant (X^2^ = 63.48, I^2^ = 87%, *P*<0.00001), and the combined effect size was not significant (SMD: -0.07, 95% CI: -0.07–0.33, *P*=0.75) (**Supplementary Figure S3**); however, the heterogeneity test result for DHEA levels in male individuals showed significant heterogeneity (X^2^ = 50.54, I^2^ = 86%, *P*<0.00001) and a significant combined effect size (SMD: 0.45, 95% CI: 0.02–0.88, *P*=0.04), indicating that males with ASD had higher DHEA levels than the control groups (**Supplementary Figure S4**). The heterogeneity test results and combined effect sizes for both TT and DHEA levels in females were not significant (**Supplementary Figures S3**, **S4**).

#### Subgroup analysis by age

When prepubertal and postpubertal studies were compared, the heterogeneity test result for prepubertal TT levels was found to be significant (X^2^ = 56.38, I^2^ = 93%, *P*<0.00001), whereas the combined effect size was not significant (SMD: 0.32, 95% CI: -0.52–1.15, *P*=0.46). The heterogeneity test result for postpubertal TT levels was significant (X^2^ = 34.04, I^2^ = 79%, *P*=0.52), but the combined effect size was not significant (SMD: -0.13, 95% CI: -0.51–0.26, *P*=0.52). Moreover, the heterogeneity test result for DHEA levels before and after puberty was significant, while the combined effect size was not (**Supplementary Figures S5**, **S6**).

#### Subgroup analysis by sex and age

We then compared the studies considering age and sex as double confounders. The heterogeneity test result for TT levels in prepubertal males was significant (X^2^ = 29.51, I^2^ = 90%, *P*<0.00001), and the combined effect size was not significant (SMD: -0.05, 95% CI: -0.84–0.73, *P*=0.90). The heterogeneity test for postpubertal male TT levels showed a significant result (X^2^ = 28.44, I^2^ = 79%, *P*<0.0001), but the combined effect size was not significant (SMD: -0.22, 95% CI: -0.62–0.17, *P*=0.26). Similarly, the heterogeneity test result for DHEA levels in prepubertal male individuals showed a significant result (X^2^ = 44.96, I^2^ = 89%, *P*<0.00001), and the combined effect size was not significant (SMD: 0.48, 95% CI: -0.03–0.99, *P*=0.07). The heterogeneity test for DHEA levels in postpubertal males yielded a significant result (X^2^ = 4.30, I^2^ = 77%, *P*=0.04), but the combined effect size was not significant (SMD: 0.03, 95% CI: -0.43–1.02, *P*=0.42). However, after grouping by age and sex, the overall heterogeneity test for DHEA levels showed significance (X^2^ = 50.18, I^2^ = 86%, *P*<0.00001), and the total combined effect size was also significant (SMD: 0.43, 95% CI: 0.02–0.84, *P*=0.04) (**Supplementary Figures S7**, **S8**).

#### Subgroup analysis by measurement method

Finally, the hormone levels detected by different methods of studies were compared. The TT levels were measured using radioimmunoassay (RIA). The heterogeneity test showed a significant difference (X^2^ = 38.77, I^2^ = 97%, *P*<0.00001). The combined effect size was not significant (SMD: 0.57, 95% CI: -0.10–1.89, *P*=0.65). The heterogeneity test for TT levels measured using enzyme-linked immunosorbent assay (ELISA) showed a significant difference (X^2^ = 50.20, I^2^ = 94%, *P*<0.00001), but the combined effect size was not significant (SMD: -0.03, 95% CI: -0.77–0.71, *P*=0.94). However, when TT levels were measured using gas chromatography–tandem mass spectrometry (GC-MS/MS), the heterogeneity test showed no significant difference (X^2^ = 0.34, I^2^ = 0%, *P*=0.56), and the combined effect was significant (SMD: 0.55, 95% CI: 0.17–0.92, *P*=0.004). In addition, when the DHEA levels were measured using RIA, the combined effect size was not significant (SMD: -0.51, 95% CI: -1.26–0.25, *P*=0.19). However, when ELISA was used to measure DHEA levels, the total heterogeneity test showed no significant difference (X^2^ = 0.78, I^2^ = 0%, *P*=0.38), and the total combined effect was significant (SMD: 1.17, 95% CI: 0.86–1.47, *P*<0.00001) (**Supplementary Figures S9**, **S10**).

### Sensitivity and publication bias

For the sensitivity analysis of 17 studies and 37 data points, deleting any literature had no great impact on the results, proving that the meta-analysis results were relatively stable. The funnel plot was nearly symmetric (**Supplementary Figures S11**, **S12**). Begg’s test showed no significant publication bias (Begg’s test, *P*=0.847, 0.675).

### Meta-regression analysis

Meta-regression analyses did not show any significant modifying effect of the major confounding factors, namely, publication year, country of origin, sample source, sex, mean age, and assay, on the observed associations (*P*=0.053–0.977; **Supplementary Table S4**).

## Discussion

In this study, we conducted a meta-analysis of 17 articles extracted from PubMed, Embase, Cochrane, Web of Science, and other databases, including 1,494 individuals, to evaluate the androgen levels in individuals with ASD compared to control groups, as well as to explore the influences of different sample sources and analytical methods on the results of TT and DHEA level measurement. After calculating the total combined effect size for TT, FT, DHEA, DHEA-S, androstenedione, androstenediol, and 5α-dihydrotestosterone levels, we found that the androgen levels in individuals with ASD were higher than those in the control groups. Sensitivity analysis showed that the results were not influenced by specific studies. Additionally, there was no significant publication bias, but there was a high degree of heterogeneity. Therefore, the results should be interpreted with caution. Androgen levels vary significantly according to age and sex, which adds complexity to our study of androgen levels in ASD. However, before drawing a conclusion, we also made a relatively sufficient assessment. First, the inclusion criteria of the study strictly required sex and age matching between the experimental group and the control group. Second, the articles included in this study were based on conclusions drawn from age- and sex-specific laboratory reference ranges for androgen metabolites relative to LabCorp. This result is consistent with that of most studies. Evidence of hyperandrogenism in individuals diagnosed with ASD is supported by multiple studies in the areas of psychological framework, brain pathology, tissue culture, and pre- and postnatal androgen levels (31, 32). We found that the increase in androgen levels was most representative of the increase in FT level, which was significantly higher in individuals with ASD than in the control groups. Previous studies have supported the idea that FT levels are elevated in people with ASD and that this elevation is associated with autistic traits (33). Findings at different developmental stages suggest that the association between FT and autistic traits is robust (34). However, notably, the analysis of FT was performed based on four studies; therefore, sensitivity analysis was not conducted, and the heterogeneity was significant.

There was a high degree of heterogeneity in current study, and subgroup analysis revealed that urine TT and DHEA levels were significantly elevated in the individuals with ASD compared to controls. This finding suggests that the correlation may be influenced by sample origin. However, the limitation of this analysis was that there were only two studies on urine sources; hence, the validity of this analysis may have been insufficient. This potential confounding factor needs to be considered in future analyses of androgen levels in individuals with ASD.

The DHEA levels measured in individuals with ASD were significantly increased in males but no enough evidence in females. In our meta-analysis, most studies documented DHEA levels in males with ASD; therefore, there are sufficient data to conclude that DHEA levels are elevated in men with ASD. However, few studies have documented DHEA levels in females with ASD. DHEA is a neuroactive neurosteroid (35), and together with other GABAergic neuroactive steroids, it is thought to influence memory and affective behaviours during adult life and with aging (36). DHEA has been shown to influence psychopathology-related processes by modulating presynaptic and postsynaptic neurotransmitter receptors (37, 38). Moreover, sociobiologists and paediatric endocrinologists have speculated on its potential role in shaping brain development before and after birth and in determining cognitive, behavioural, and sexual orientation in children (39). Many studies have reported abnormal serum DHEA concentrations in individuals with major neurodevelopmental and neurodegenerative diseases, such as schizophrenia, bipolar disorder, depression, and Alzheimer’s disease (40). However, the mechanistic evidence for a clear role of DHEA in any of these areas of human development remains speculative (41). Therefore, our meta-analysis speculated that elevated DHEA levels in individuals with ASD might reflect the effects on cognition, memory, neurodevelopment, and behaviour. This effect is more obvious in men with ASD; however, because only few studies have analysed DHEA levels in women, it is necessary to further study the DHEA levels in this population.

In addition, when measured using GC-MS/MS, the TT levels in individuals with ASD were higher than those in the control groups, but the DHEA levels were not. When measured using ELISA, the DHEA levels, but not TT levels, were higher in individuals with ASD than in control groups, suggesting that different methods for hormone level measurement may affect the result. One intriguing hypothesis is that ASD is a ‘whole-body disorder’ (42), involving metabolic pathways that are expressed across the whole body (43). A low level of cholesterol—the ‘precursor’ of all the steroid hormones, with the exception of VitD—has been indirectly linked to autism in a variety of studies of autism-associated medical syndromes, ranging from Smith–Lemli–Opitz syndrome (44) to the Fragile‐X‐syndrome (45). Steroid hormones are cholesterol-derived molecules that regulate a wide range of human activities. Steroids can be classified into two different groups: corticosteroids and sex steroids. Sex steroids are responsible to maintain pregnancy (progestogens) and to develop and preserve male (androgens) and female (oestrogens) sexual characteristics. At present, immunoassays as well as mass spectrometric methods are common for measuring steroid hormone levels in clinical and research laboratories (46). In the literature included in this study, androgen level measurement was performed using these two commonly used methods. In general, immunoassays (whether RIA or ELISA based) have drawbacks of cross-reactivity and lack of specificity, particularly for closely related steroid metabolites (47). However, using GC-MS/MS, different steroids are separated by the chromatographic procedure, and their identity is confirmed via mass spectrometry. Therefore, appropriate measurement methods should be selected according to the characteristics of hormone analysis. Additionally, differences in the timing of sample collection may affect the results of hormone level measurement.

The mechanism of androgen level elevation in individuals with ASD remains unclear. The extreme male brain (EMB) theory of ASD has been vigorously debated since proposed by Baron-Cohen over 20 years ago, which is based on the concept of empathy and systematisation, namely, sex-differentiated behaviour (48). This theory assumes that individuals with ASD have an EMB with an extreme systematic behaviour pattern. However, the specific reasons for the differences in brain development in individuals with ASD are still being studied (49). It has been demonstrated decreased aromatase levels in the frontal cortex of individuals diagnosed with ASD and a positive correlation between aromatase levels and the protein product of the ASD-associated gene RORA (retinoic acid-related orphan receptor-alpha) (50). Therefore, genes associated with ASD may be modulated by sex hormones, and the enzyme aromatase may be a critical factor in the overrepresentation of males with ASD. In addition, the ASD-related androgen or foetal testosterone theory (51, 52) suggests that utero exposure to higher levels of testosterone affects the development of the foetal brain and leads to a systematic behaviour, consequently resulting in ASD. The theory postulates that higher levels of testosterone during critical periods can influence early brain development, inducing organisational effects on the brain structure that influence subsequent behaviour. A Meta-analysis reported that mothers with PCOS had a significantly increased risk of ASD in their offspring (53). Our study suggests that the increased androgen level in individuals with ASD may provide a reference value for clinical research in the future.

## Strengths and limitations

The limitations of our study are as follows. First, we did not analyse some of the clinical confounding factors, such as BMI, country, race, intake of relevant drugs, the individuals’ condition at birth, and the mother’s information. Environmental risk factors may not only increase the risk of ASD through several complex underlying mechanisms, such as genetic and epigenetic effects, inflammation and oxidative stress, or hypoxia and ischaemic injury (54), but also affect early development and androgen levels in individuals with ASD. Second, the sample size of female individuals with ASD was insufficient. Most of the included studies had assessed male individuals with ASD, and there were only few studies on female individuals with ASD. Third, there was a relatively high heterogeneity, but the source of heterogeneity could not be well explained after subgroup analysis. The random-effects model was used, which may be related to different methods and measurement units in androgen level measurement, thereby greatly affecting the accuracy of the results.

The strengths of this study are as follows. First, there was an extensive collection of studies from multiple databases, and references were searched in the literature. The recall rate was high, and only case-control studies were included. Upon quality evaluation, the literature quality was found to be high. Second, during sensitivity analysis, there was no significant impact on the results during exclusion of any study, indicating that the results were relatively stable. Finally, no obvious publication bias existed after bias testing, and the results were highly reliable.

## Conclusion

In conclusion, despite these limitations, our current findings suggest that androgen levels are altered in individuals with ASD. The main analysis shows that androgen and FT levels are significantly elevated in individuals with ASD compared with control, and the secondary analysis shows that DHEA level is significantly elevated in male with ASD compared with control. These results are of clinical significance for early identification and evaluation of ASD. Currently, in the absence of treatment, ASD is often regarded a lifelong developmental disorder that profoundly affects the way a person understands, communicates, and relates to others. The androgen level is an important characteristic in individuals with ASD, and many investigations have shown that the clinical characteristics in individuals with ASD are significantly correlated with androgen hypersecretion. Changes in the TT and DHEA levels in these individuals remain ambiguous. Therefore, in future studies on ASD, more attention should be paid to the changes in FT levels, and additional comprehensive studies on TT and DHEA levels in all individuals with ASD, particularly, female individuals, should be conducted to confirm the relationship between ASD and androgens.

## Data availability statement

The original contributions presented in the study are included in the article/**Supplementary Material**. Further inquiries can be directed to the corresponding authors.

## Author contributions

SL: Funding acquisition, Writing – review & editing. ZW: Writing – original draft. BZ: Writing – original draft. CM: Writing – original draft. DQ: Writing – original draft. HC: Writing – original draft. YZ: Writing – original draft. HXC: Writing – original draft. RZ: Writing – review & editing.
