# Supplementary material for: Androgen levels in autism spectrum disorders: a systematic review and meta-analysis
Source: Front Endocrinol (Lausanne). 2024 May 8;15:1371148. doi: 10.3389/fendo.2024.1371148 (PMC11109388; doi:10.3389/fendo.2024.1371148)
Supplement: Supplementary file 1 [file Table_1.docx]

**Table S1：**Characteristics of the included studies.

| Study | Country of recruitment | Individuals with ASD | | | |  | Controls | | | Illness | Biological  material | Hormones | Assay | Medication status | NOS |
| --- | --- | --- | --- | --- | --- | --- | --- | --- | --- | --- | --- | --- | --- | --- | --- |
|  |  | n (M/F) | Age, years ^a^ | Source of ASD | criteria for diagnosing |  | n (M/F) | Age, years ^a^ | Source of controls |  |  |  |  |  |  |
| Ruta et al. (2011) | UK | 58 (25/33) | 31.48 ± 8.32 | The Cambridge Autism Research Centre database of volunteers、the National Autistic Society (UK),and local autism support groups in the UK | DSM-IV-TR |  | 70 (31/39) | 31.43 ± 7.45 | Advertising to recruit | Asperger’s syndrome | Serum | Total/Free testosterone  Androstenedione  DHEA-S | RIA^c^ Siemens IMMULITE 1000^d^ | Excluded | 9 |
| Croonenberghs et al. (2010) | Belgium | 18 (18/0) | 12-18 | The outpatient clinic of University Child and Adolescent Psychiatry in Antwerp, Belgium | ADI-R DSM-IV |  | 22 (22/0) | 12-18 | ns | Autism | Serum | Total testosterone | ns | ns | 8 |
| Tordjman et al. (1995) | UK | 51 (51/0) | 8.38 ± 6.95 | Special schools | DSM-III-R |  | 21 (21/0) | 15.24 ± 8.59 | Local schools and news- papers | Autism | Plasma | Total testosterone DHEA-S | RIA | Excluded | 9 |
| Majewska et al. (2014) | Poland | 78 (43/35) | 5.57 ± 2.23 | In out-patient clinics | DSM IV |  | 70 (36/34) | 5.93 ± 3.36 | Six preschools and three primary Warsaw schools | Autism | Saliva | DHEA DHEA-S Androstenediol Androstenedione | GC-MS/MS RIA | ns | 9 |
| Janšáková et al. (2020) | Slovakia | 62 (62/0) | 4.40 ± 1.10 | ns | DSM V |  | 24 (24/0) | 4.29 ± 0.95 | ns | ASD | Plasma | DHEA DHEA-S Androstenediol | GC-MS/MS | ns | 7 |

**Table 1.** (continue)

| Study | Country of recruitment | Individuals with ASD | | | |  | Controls | | | Illness | Biological  material | Hormones | Assay | Medication status | NOS |
| --- | --- | --- | --- | --- | --- | --- | --- | --- | --- | --- | --- | --- | --- | --- | --- |
|  |  | n (M/F) | Age, years ^a^ | Source of ASD | criteria for diagnosing |  | n (M/F) | Age, years ^a^ | Source of controls |  |  |  |  |  |  |
| Gasser et al. (2019) | Austria | 41 (41/0) | 14.40 ± 3.30 | The area of Leipzig (Austria) | DSM V |  | 41 (41/0) | 13.70 ± 2.80 | The area of Leipzig (Austria) | Asperger’s syndrome | Urine | Total testosterone 5α-Dihydrotestosterone  DHEA Androstenediol | GC-MS/MS | ns | 9 |
| Schmidtova et al. (2010) | Slovakia | 101 (101/0) | 4-18 | ns | DSM-IV |  | 107 (107/0) | 4-18 | ns | Autism Asperger’s syndrome | Saliva | Testosterone | ELISA | ns | 7 |
| Sharpley et al. (2017) | Australia | 136 (136/0) | 11.30 ± 3.20 | Gold Coast, Queensland, Australia | ADOS |  | 48 (48/0) | 11.80 ± 3.10 | Local schools | ASD | Saliva | Total testosterone | ELISA | ns | 8 |
| Al-Zaid et al. (2014) | Saudi Arabia | 31 (31/0) | 5.60 ± 0.31 | Autism Research and Treatment Center, College of Medicine, King Saud University, Riyadh, Saudi Arabia | DSM IV |  | 28 (28/0) | 5.44 ± 0.23 | ns | Autism | Plasma | Total testosterone  Free testosterone | ELISA | ns | 8 |
| Gasser et al. (2020) | Austria | 16 (0/16) | 14.30 ± 4.20 | The area of Leipzig (Austria) | DSM-IV |  | 16 (0/16) | 14.40 ± 4.00 | The area of Leipzig (Austria) | Autism | Urine | Total testosterone Androstenediol 5a-Dihydrotestosteron DHEA | GC-MS/MS | ns | 8 |
| Chew et al. (2021) | UK | 21 (21/0) | 23.71 ± 5.05 | ns | DSM-V |  | 20 (20/0) | 26.00 ± 6.69 | ns | ASD | Serum and saliva | Total testosterone DHEA | LC-MS/MS | ns | 9 |

**Table 1.** (continue)

| Study | Country of recruitment | Individuals with ASD | | | |  | Controls | | | Illness | Biological  material | Hormones | Assay | Medication status | NOS |
| --- | --- | --- | --- | --- | --- | --- | --- | --- | --- | --- | --- | --- | --- | --- | --- |
|  |  | n (M/F) | Age, years ^a^ | Source of ASD | criteria for diagnosing |  | n (M/F) | Age, years ^a^ | Source of controls |  |  |  |  |  |  |
| Mills et al. (2007) | UK | 71 (71/0) | 6.60 ± 1.50 | At Cincinnati Children’s Hospital Medical Center (CCHMC) | DSM-IV |  | 59 (59/0) | 6.50 ± 1.20 | CCHMC’s ENT outpatient | ASD | Plasma | Total testosterone DHEA | ns | Excluded | 8 |
| El-Baz et al. (2014) | Egypt | 30 (30/0) | 9.13 ± 2.36 | Pediatrics Psychiatry Clinic, Children’s Hospital | DSM IV |  | 20 (20/0) | 9.45 ± 2.47 | pediatrics outpatient clinic of the same hospital | Autism | Serum | Total testosterone DHEA | ELISA | Excluded | 9 |
| Strous et al. (2005) | Israel | 15 (11/4) | 23.60 ± 4.20 | ns | DSM-IV |  | 13 (6/7) | 30.30 ± 4.10 | ns | Autism | Serum | DHEA DHEA-S | RIA | Excluded | 7 |
| Hassan et al. (2019) | Egypt | 73 (73/0) | 7.13 ± 3.52 | Outpatient psychiatric clinics of the Neuropsychiatric and Pediatric Departments of the University Hospitals | CARS |  | 73 (73/0) | 7.76 ± 4.37 | Outpatient psychiatric clinics | Autism | Serum | Free testosterone DHEA | ELISA | Excluded | 9 |
| Krajmer et al. (2011) | Slovakia | 50（50/0） | 8.32^e^  13.71^f^ | The local centers in various towns of Slovak Republic | DSM-IV |  | 79（79/0） | 9.10^e^ 14.83^f^ | Elementary and grammar schools | Asperger’s syndrome | Saliva | Total testosterone | ELISA | ns | 8 |
| Ma et al. (2020) | China | 35 (22/13) | 4.05 ± 1.16 | Children with autism who have been clearly diagnosed and trained in Luohe Rehabilitation Center for the Disabled | DSM-V |  | 40 (25/15) | 4.13 ± 1.07^b^ | Luohe Public Kindergarten | Autism | Serum | Total testosterone | RIA | ns | 8 |

Abbreviations:

RIA, radioimmunoassay; GC-MS/MS, gas chromatography–mass spectrometry; LC-MS/MS, liquid chromatography-tandem mass spectrometry; ELISA, enzyme linked immunosorbent assay; DHEA, Dehydroepiandrosterone; DHEA-S, Dehydroepiandrosterone Sulfate; DSM-IV-TR, Diagnostic and Statistical Manual-IV-Text Review Disorders criteria; ADI-R, Autism Diagnostic Interview-Revised; DSM-IV, Diagnostic and Statistical Manual-IV; CARS, Childhood Autism Rating Scale; ADOS, Autism Diagnostic Observation Schedule; ICD-10, International Classification of Diseases, 10th edition; NOS, Newcastle-Ottawa Scale; ns, not specified.

^a^ Data expressed as mean ±SD or range or mean.

^b^ SD calculated according to the formula: Mean = month/12, SD = month/12.

^c^ Total testosterone was measured by RIA.

^d^ Androstenedione and DHEA-S were measured by Siemens IMMULITE 1000.

^e^ Prepubertal (under the age of ten, including ten years old), mean

^f^ Postpubertal (more than ten years old), mean
