# Supplementary material for: Androgen levels in autism spectrum disorders: a systematic review and meta-analysis
Source: Front Endocrinol (Lausanne). 2024 May 8;15:1371148. doi: 10.3389/fendo.2024.1371148 (PMC11109388; doi:10.3389/fendo.2024.1371148)
Supplement: Supplementary file 2 [file Table_2.docx]

**Table S2** The levels of androgen in individuals with ASD as well as control groups.

Abbreviations:

TT, total testosterone; FT, free testosterone; DHEA, dehydroepiandrosterone; DHEA-s, dehydroepiandrosterone sulfate; 5α-DHT, 5α-Dihydrotestosterone; ASD, Autism spectrum Disorder; AS, Asperger syndrome; UK, unknown.
