# Supplementary material for: Androgen levels in autism spectrum disorders: a systematic review and meta-analysis
Source: Front Endocrinol (Lausanne). 2024 May 8;15:1371148. doi: 10.3389/fendo.2024.1371148 (PMC11109388; doi:10.3389/fendo.2024.1371148)
Supplement: Supplementary file 3 [file Table_3.docx]

**Table S3** Results of quality assessment using the Newcastle-Ottawa Scale for case-control studies

| Study | Selection | | | |  | Comparability |  | Exposure | | | Scores |
| --- | --- | --- | --- | --- | --- | --- | --- | --- | --- | --- | --- |
|  | Adequate definition of cases | Representativeness of the cases | Selection of ontrols | Definition of controls |  | Control for important factors^a^ |  | Ascertainment of exposure | Same method of ascertainment for cases and controls | Non response rate |  |
| Ruta, L 2011 | ☆ | ☆ | ☆ | ☆ |  | ☆☆ |  | ☆ | ☆ | ☆ | 9 |
| Croonenberghs, J 2010 | ☆ | ☆ | – | ☆ |  | ☆☆ |  | ☆ | ☆ | ☆ | 8 |
| Tordjman, S 1995 | ☆ | ☆ | ☆ | ☆ |  | ☆☆ |  | ☆ | ☆ | ☆ | 9 |
| Majewska, M.D 2014 | ☆ | ☆ | ☆ | ☆ |  | ☆☆ |  | ☆ | ☆ | ☆ | 9 |
| Janšáková, K 2020 | – | ☆ | – | ☆ |  | ☆☆ |  | ☆ | ☆ | ☆ | 7 |
| Gasser, B.A 2019 | ☆ | ☆ | ☆ | ☆ |  | ☆☆ |  | ☆ | ☆ | ☆ | 9 |
| Schmidtova, E 2010 | – | ☆ | – | ☆ |  | ☆☆ |  | ☆ | ☆ | ☆ | 7 |
| Sharpley, C. F 2017 | ☆ | ☆ | ☆ | – |  | ☆☆ |  | ☆ | ☆ | ☆ | 8 |
| Al-Zaid, F. S 2014 | ☆ | ☆ | – | ☆ |  | ☆☆ |  | ☆ | ☆ | ☆ | 8 |
| Gasser, B. A 2020 | ☆ | ☆ | ☆ | – |  | ☆☆ |  | ☆ | ☆ | ☆ | 8 |
| Chew, L 2021 | ☆ | ☆ | ☆ | ☆ |  | ☆☆ |  | ☆ | ☆ | ☆ | 9 |
| Mills, J. L 2007 | ☆ | ☆ | – | ☆ |  | ☆☆ |  | ☆ | ☆ | ☆ | 8 |
| El-Baz, F 2014 | ☆ | ☆ | ☆ | ☆ |  | ☆☆ |  | ☆ | ☆ | ☆ | 9 |
| Strous, R. D 2005 | ☆ | ☆ | ☆ | ☆ |  | – |  | ☆ | ☆ | ☆ | 7 |
| Hassan, M. H 2019 | – | ☆ | ☆ | ☆ |  | ☆☆ |  | ☆ | ☆ | ☆ | 9 |
| Krajmer, P 2011 | ☆ | ☆ | ☆ | ☆ |  | ☆ |  | ☆ | ☆ | ☆ | 8 |
| Ma, F 2020 | ☆ | ☆ | ☆ | ☆ |  | ☆ |  | ☆ | ☆ | ☆ | 8 |
| ^a^，a maximum of 2 stars can be allotted in this category, one for age and gender, the other for other controlled factors; -, On behalf of the zero. | | | | | | | | | | | |
