# Supplementary material for: Androgen levels in autism spectrum disorders: a systematic review and meta-analysis
Source: Front Endocrinol (Lausanne). 2024 May 8;15:1371148. doi: 10.3389/fendo.2024.1371148 (PMC11109388; doi:10.3389/fendo.2024.1371148)
Supplement: Supplementary file 4 [file Table_4.docx]

**Table S4** Meta-regression of androgen levels.

Abbreviation：

Adj.R2–proportion of between-study variance that can be explained by the model,

N–number of obs, TT–total testosterone, DHEA – dehydroepiandrosterone.
