# Supplementary material for: Androgen levels in autism spectrum disorders: a systematic review and meta-analysis
Source: Front Endocrinol (Lausanne). 2024 May 8;15:1371148. doi: 10.3389/fendo.2024.1371148 (PMC11109388; doi:10.3389/fendo.2024.1371148)
Supplement: Supplementary file 5 [file Table_5.docx]

**Table S5** Search strategies

644 of PubMed

| Search number | Query | Results |
| --- | --- | --- |
| #1 | "autism*"[All Fields] OR "autistic"[All Fields] OR "Early Infantile Autism"[All Fields] OR "autism early infantile"[All Fields] OR "Infantile Autism"[All Fields] OR "Autism Spectrum Disorder"[All Fields] OR "ASD"[All Fields] OR "kanner syndrome"[All Fields] OR "Fragile X Syndrome"[All Fields] OR "FRAXA Syndrome"[All Fields] OR "FRAXE Syndrome"[All Fields] OR "fra x syndrome"[All Fields] OR "Fragile X Mental Retardation Syndrome"[All Fields] OR "mar x syndrome"[All Fields] OR "Marker X Syndrome"[All Fields] OR "X Linked Mental Retardation and Macroorchidism"[All Fields] OR "Martin Bell Syndrome"[All Fields] OR "asperger syndrome"[All Fields] OR "asperger disorder"[All Fields] | 95,982 |
| #2 | "androgen"[All Fields] OR "Androstenedione"[All Fields] OR "4-Androstene-3,17-dione"[All Fields] OR "Testosterone"[All Fields] OR "Testolin"[All Fields] OR "Epitestosterone"[All Fields] OR "17-alpha-Testosterone"[All Fields] OR "Dihydrotestosterone"[All Fields] OR "Dehydroepiandrosterone"[All Fields] OR "5 alpha-Dihydrotestosterone"[All Fields] OR "5alpha-DHT"[All Fields] OR "DHT"[All Fields] OR "TST"[All Fields] OR "DHEA"[All Fields] OR "DHEA-S"[All Fields] | 211,480 |
| #3 | #1 AND #2 | 644 |

465 of Embase

| NO. | Query | Results |
| --- | --- | --- |
| #1 | 'autism':ti,ab,kw OR 'autistic':ti,ab,kw OR 'autism, early infantile':ti,ab,kw OR 'early infantile autism':ti,ab,kw OR 'infantile autism':ti,ab,kw OR 'autism spectrum disorder':ti,ab,kw OR 'kanners syndrome':ti,ab,kw OR 'kanner syndrome':ti,ab,kw OR 'fragile x syndrome':ti,ab,kw OR 'fraxa syndrome':ti,ab,kw OR 'fraxe syndrome':ti,ab,kw OR 'fra(x) syndrome':ti,ab,kw OR 'fragile x mental retardation syndrome':ti,ab,kw OR 'mar (x) syndrome':ti,ab,kw OR 'marker x syndrome':ti,ab,kw OR 'x linked mental retardation and macroorchidism':ti,ab,kw OR 'martin bell syndrome':ti,ab,kw OR 'asperger syndrome':ti,ab,kw OR 'asperger disorder':ti,ab,kw OR 'aspergers disorder':ti,ab,kw OR 'aspergers syndrome':ti,ab,kw | 93,138 |
| #2 | 'androgen':ti,ab,kw OR 'androstenedione':ti,ab,kw OR '4-androstene-3,17-dione':ti,ab,kw OR 'testosterone':ti,ab,kw OR 'testolin':ti,ab,kw OR 'epitestosterone':ti,ab,kw OR '17-alpha-testosterone':ti,ab,kw OR 'dihydrotestosterone':ti,ab,kw OR 'dehydroepiandrosterone':ti,ab,kw OR '5 alpha-dihydrotestosterone':ti,ab,kw OR '5α-dht':ti,ab,kw OR 'dht':ti,ab,kw OR 'tst':ti,ab,kw OR 'dhea':ti,ab,kw OR 'dhea-s':ti,ab,kw | 218,845 |
| #3 | #1 AND #2 | 465 |

30 of The Cochrane Library

| ID | Search | Hits |
| --- | --- | --- |
| #1 | "Autism":ti,ab,kw OR "autistic":ti,ab,kw OR "Autism, Early Infantile":ti,ab,kw OR "Early Infantile Autism":ti,ab,kw OR "Infantile Autism":ti,ab,kw OR "Autism Spectrum Disorder":ti,ab,kw OR "ASD":ti,ab,kw OR "Kanner's Syndrome":ti,ab,kw OR "Kanner Syndrome":ti,ab,kw OR "Fragile X Syndrome":ti,ab,kw OR "FRAXA Syndrome":ti,ab,kw OR "FRAXE Syndrome":ti,ab,kw OR "Fra(X) Syndrome":ti,ab,kw OR "Fragile X Mental Retardation Syndrome":ti,ab,kw OR "Mar (X) Syndrome":ti,ab,kw OR "Marker X Syndrome":ti,ab,kw OR "X Linked Mental Retardation and Macroorchidism":ti,ab,kw OR "Martin Bell Syndrome":ti,ab,kw OR "Asperger Syndrome":ti,ab,kw OR "Asperger's Disorder":ti,ab,kw OR "Asperger's Syndrome":ti,ab,kw OR "Asperger Disorder":ti,ab,kw OR "Aspergers Disorder":ti,ab,kw OR "Aspergers Syndrome":ti,ab,kw | 5,821 |
| #2 | "androgen":ti,ab,kw OR "androstenedione":ti,ab,kw OR "4-Androstene-3,17-dione":ti,ab,kw OR "Testosterone":ti,ab,kw OR "Testolin":ti,ab,kw OR "Epitestosterone":ti,ab,kw OR "17-alpha-Testosterone":ti,ab,kw OR "Dihydrotestosterone":ti,ab,kw OR "Dehydroepiandrosterone":ti,ab,kw OR "5 alpha-Dihydrotestosterone":ti,ab,kw OR "5α-DHT":ti,ab,kw OR "DHT":ti,ab,kw OR "TST":ti,ab,kw OR "DHEA":ti,ab,kw OR "DHEA-S":ti,ab,kw | 15,577 |
| #3 | #1 AND #2 | 30 |

432 of Ovid

| ID | Search | Results |
| --- | --- | --- |
| #1 | (androgen or Androstenedione or 4-Androstene-3,17-dione or Testosterone or Testolin or Epitestosterone or 17-alpha-Testosterone or Dihydrotestosterone or Dehydroepiandrosterone or 5 alpha-Dihydrotestosterone or DHT or TST or DHEA or DHEA-S).af. | 77,931 |
| #2 | (Autism* or autistic or Early Infantile Autism or Autism, Early Infantile or Infantile Autism or Autism Spectrum Disorder or ASD or Kanner* Syndrome or Fragile X Syndrome or FRAXA Syndrome or FRAXE Syndrome or FraX Syndrome or Fragile X Mental Retardation Syndrome or Mar X Syndrome or Marker X Syndrome or (X Linked Mental Retardation and Macroorchidism) or Martin Bell Syndrome or Asperger* Syndrome or Asperger* Disorder).af. | 180,044 |
| #3 | #1 AND #2 | 432 |
