# Supplementary material for: Androgen levels in autism spectrum disorders: a systematic review and meta-analysis
Source: Front Endocrinol (Lausanne). 2024 May 8;15:1371148. doi: 10.3389/fendo.2024.1371148 (PMC11109388; doi:10.3389/fendo.2024.1371148)
Supplement: Supplementary file 6 [file Presentation_1.pdf]

Fig. S1 Forest plot of 11 studies comparing TT levels between individuals with ASD versus Controls: sub-grouped by sample source

Abbreviations: SMD, Standardized mean difference; CI, confidence interval; TT, Total testosterone; ASD, Autism spectrum disorder.

Fig. S2 Forest plot of 10 studies comparing DHEA levels between individuals with ASD versus Controls: sub-grouped by sample source

Abbreviations: SMD, Standardized mean difference; CI, confidence interval; DHEA, Dehydroepiandrosterone; ASD, Autism spectrum disorder.

Fig. S3 Forest plot of 11 studies comparing TT levels between individuals with ASD versus Controls: sub-grouped by sex

Abbreviations: SMD, Standardized mean difference; CI, confidence interval; TT, total testosterone; ASD, Autism spectrum disorder.

Fig. S4 Forest plot of 10 studies comparing DHEA levels between individuals with ASD versus Controls: sub-grouped by sex

Abbreviations: SMD, Standardized mean difference; CI, confidence interval; DHEA, Dehydroepiandrosterone; ASD, Autism spectrum disorder.

Fig. S5 Forest plot of 11 studies comparing TT levels between individuals with ASD versus Controls: sub-grouped by age

Abbreviations: SMD, Standardized mean difference; CI, confidence interval; TT, total testosterone; ASD, Autism spectrum disorder.

Fig. S6 Forest plot of 10 studies comparing DHEA levels between individuals with ASD versus Controls: sub-grouped by age

Abbreviations: SMD, Standardized mean difference; CI, confidence interval; DHEA, Dehydroepiandrosterone; ASD, Autism spectrum disorder.

Fig. S7 Forest plot of 11 studies comparing TT levels between individuals with ASD versus Controls: sub-grouped by sex and age

Abbreviations: SMD, Standardized mean difference; CI, confidence interval; TT, total testosterone; ASD, Autism spectrum disorder.

Fig. S8 Forest plot of 10 studies comparing DHEA levels between individuals with ASD versus Controls: sub-grouped by age

Abbreviations: SMD, Standardized mean difference; CI, confidence interval; DHEA, Dehydroepiandrosterone; ASD, Autism spectrum disorder.

Fig. S9 Forest plot of 11 studies comparing TT levels between individuals with ASD versus Controls: sub-grouped by measurement methods

Abbreviations: SMD, standardized mean difference; CI, confidence interval; TT, total testosterone; ASD, Autism spectrum disorder; RIA, radioimmunoassay; GC-MS/MS,

gas chromatography–mass spectrometry; LC-MS/MS, liquid chromatography-tandem mass spectrometry; ELISA, enzyme linked immunosorbent assay.

Fig. S10 Forest plot of 10 studies comparing DHEA levels between individuals with ASD versus Controls: measurement method

Abbreviations: SMD, standardized mean difference; CI, confidence interval; DHEA, Dehydroepiandrosterone; ASD, Autism spectrum disorder; RIA, radioimmunoassay; GC-MS/MS, gas chromatography–mass spectrometry; ELISA, enzyme linked immunosorbent assay

Fig. S11 Funnel plot of the relative TT levels in individuals with ASD

Abbreviations: SMD, standardized mean difference

Fig. S12 Funnel plot of the relative DHEA levels in individuals with ASD

Abbreviations: SMD, standardized mean difference
