# Supplementary figures and images for: Androgen levels in autism spectrum disorders: a systematic review and meta-analysis
Source: Front Endocrinol (Lausanne). 2024 May 8;15:1371148. doi: 10.3389/fendo.2024.1371148 (PMC11109388; doi:10.3389/fendo.2024.1371148)

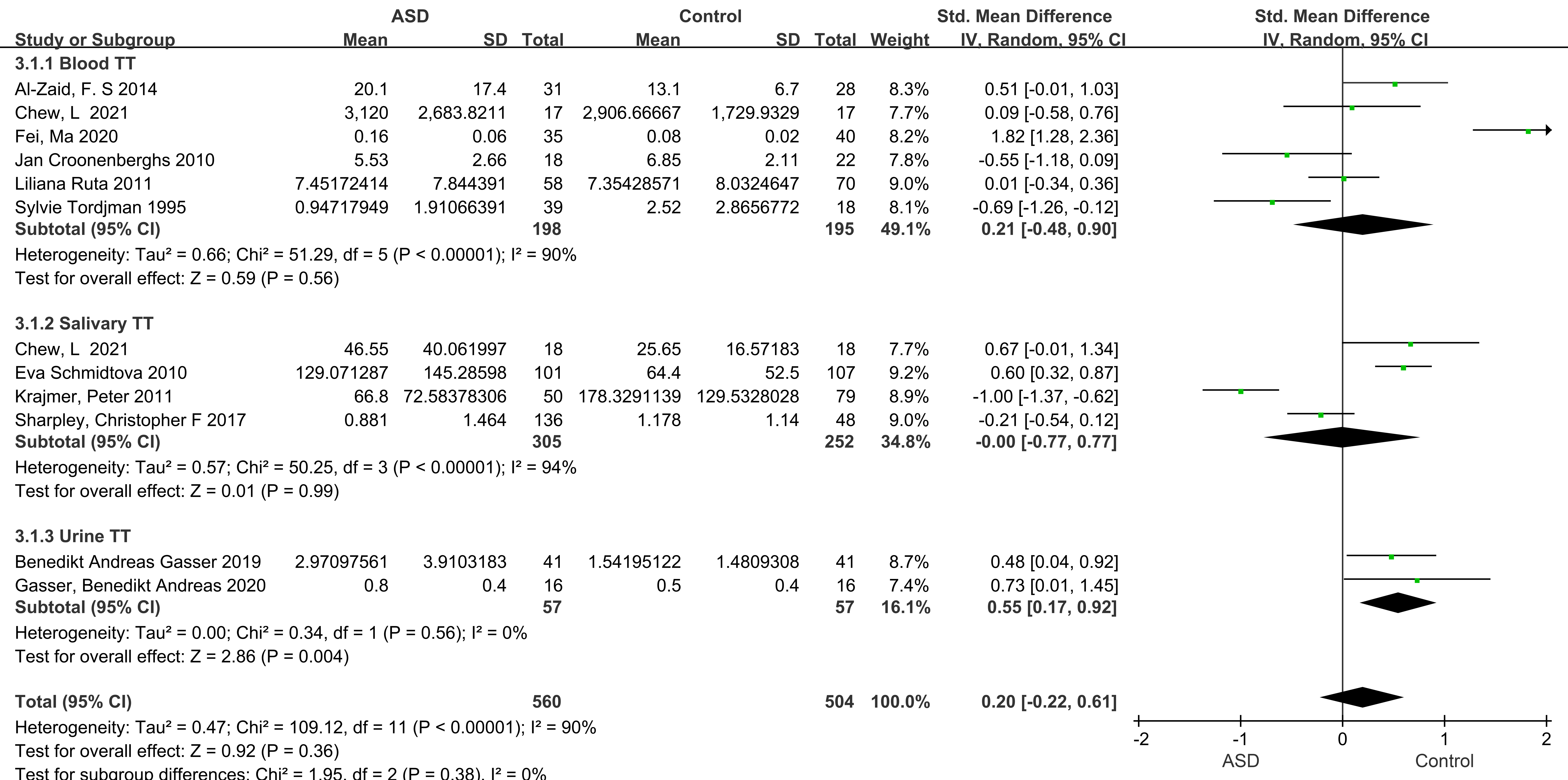

Supplement: Supplementary file 7 [file Image_1.jpeg]

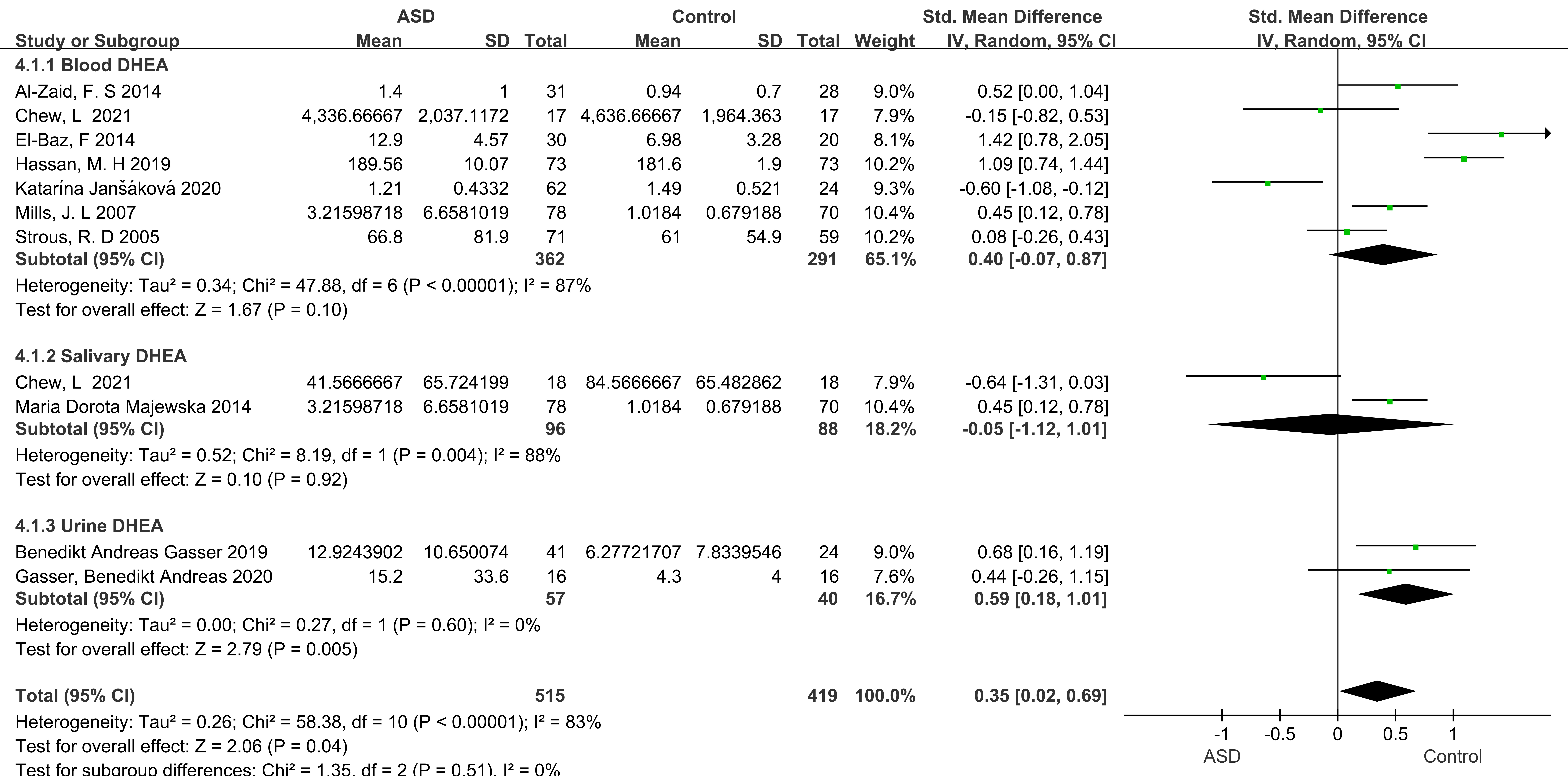

Supplement: Supplementary file 8 [file Image_2.jpeg]

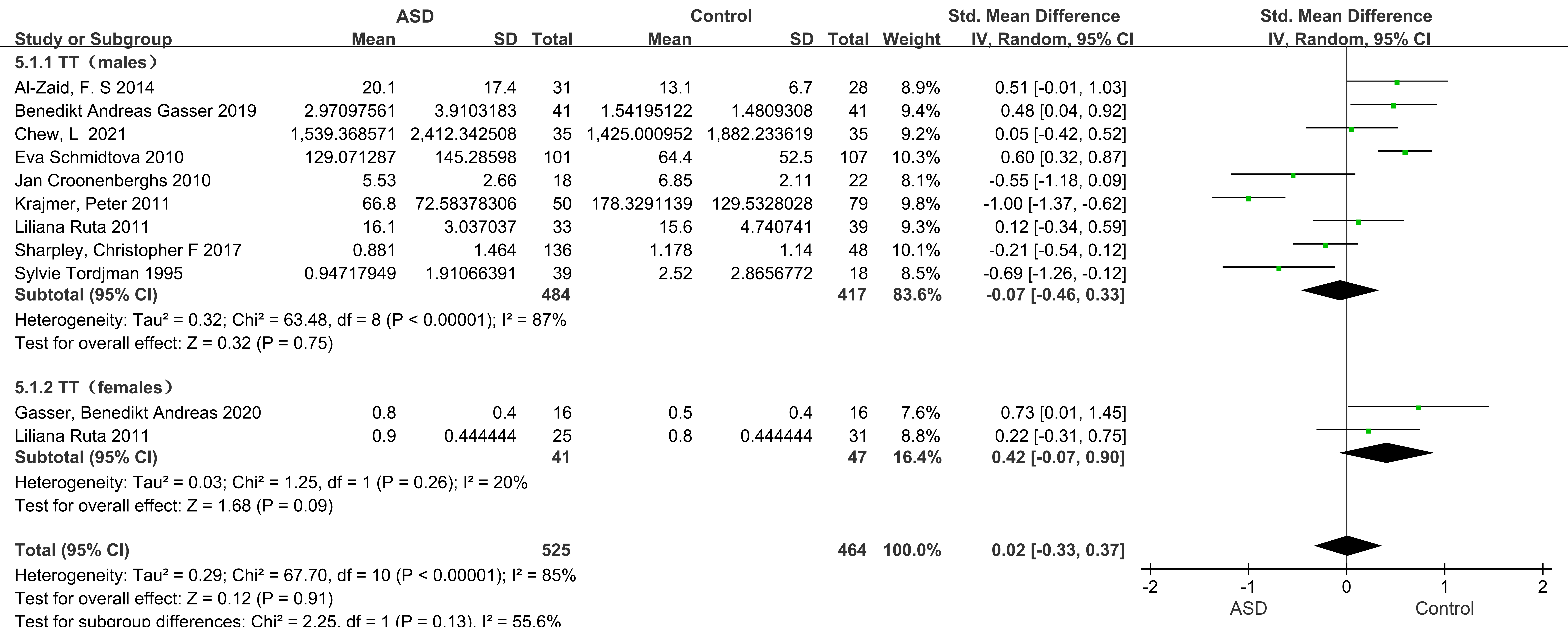

Supplement: Supplementary file 9 [file Image_3.jpeg]

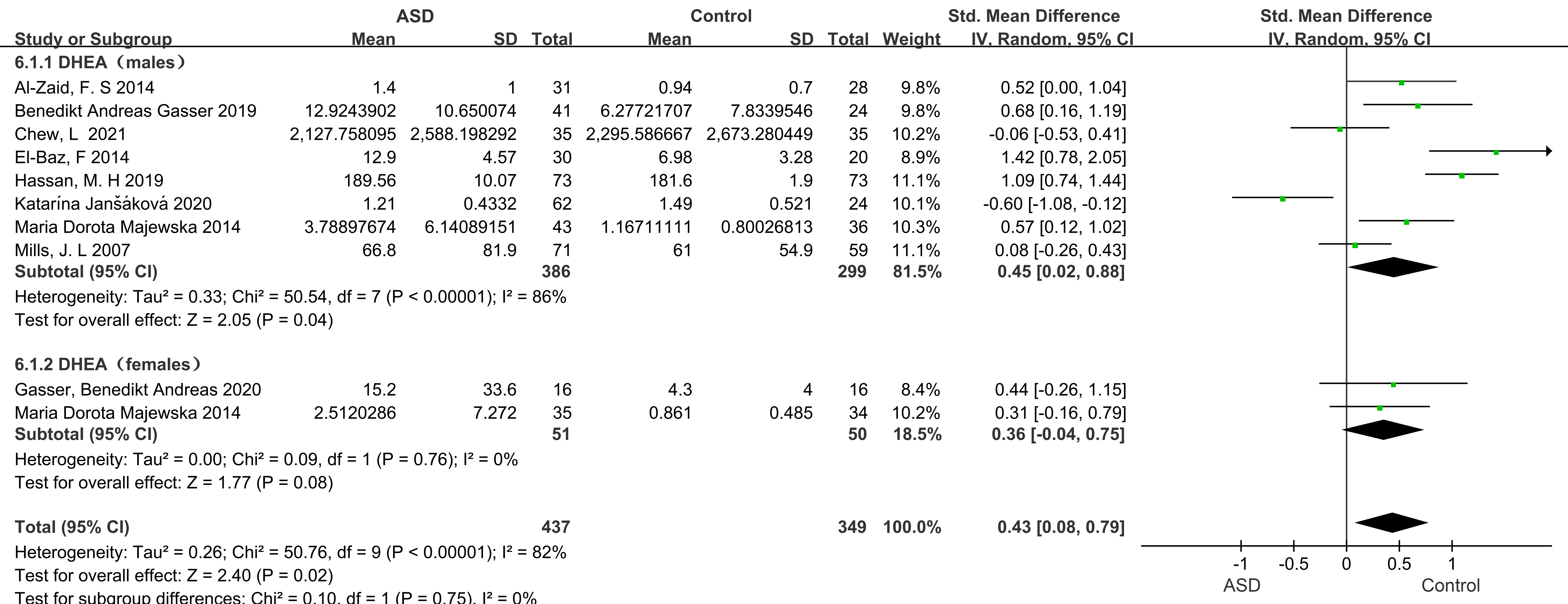

Supplement: Supplementary file 10 [file Image_4.jpeg]

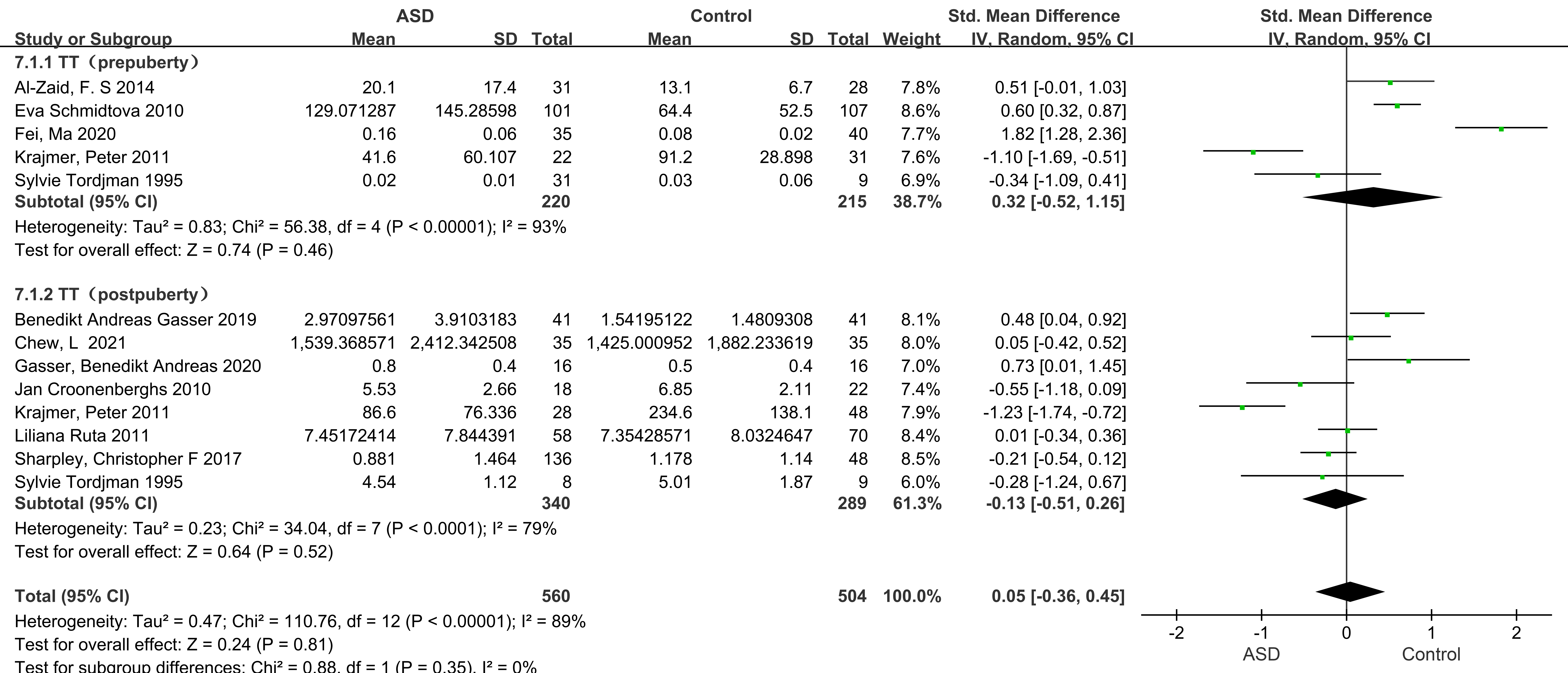

Supplement: Supplementary file 11 [file Image_5.jpeg]

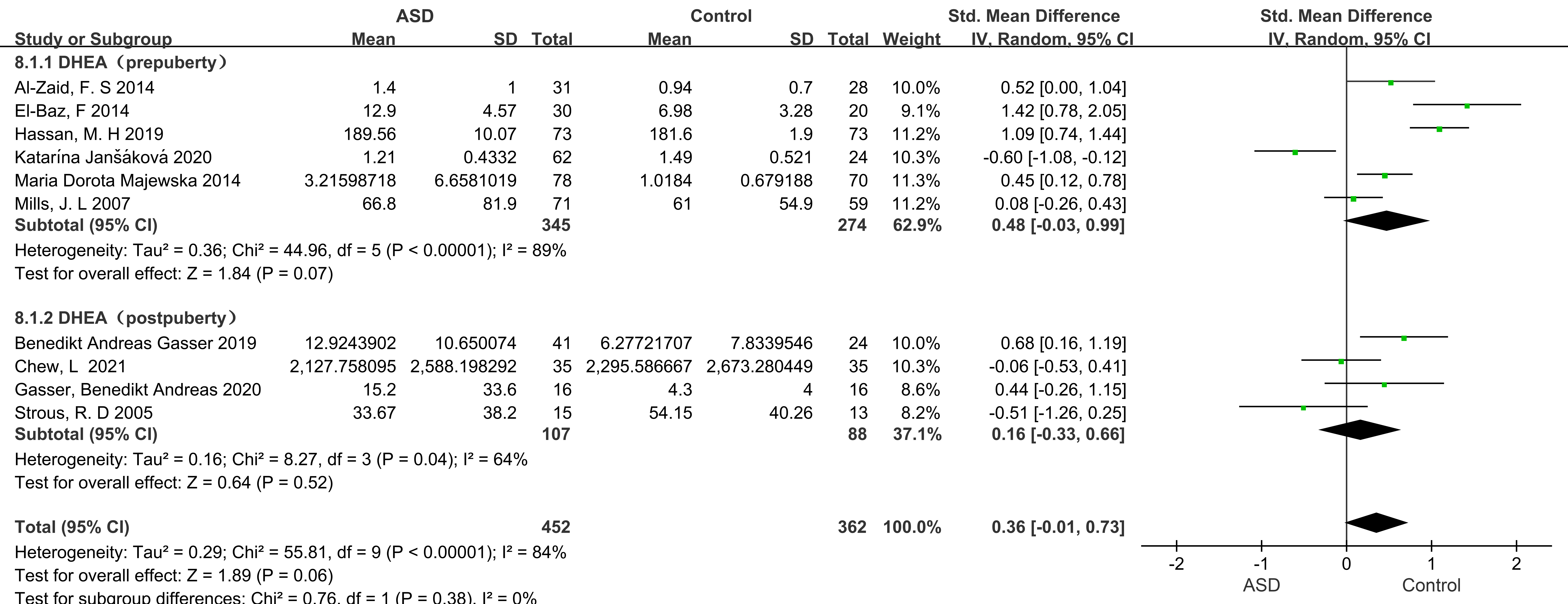

Supplement: Supplementary file 12 [file Image_6.jpeg]

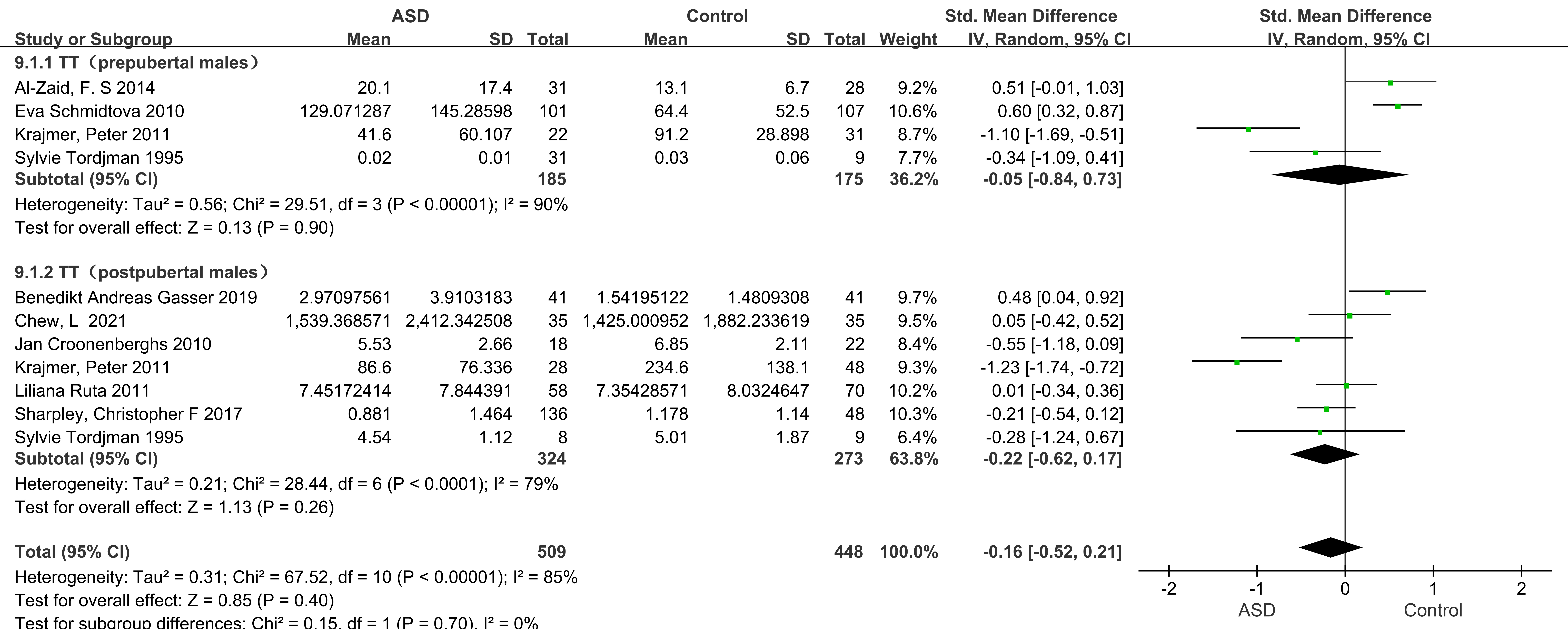

Supplement: Supplementary file 13 [file Image_7.jpeg]

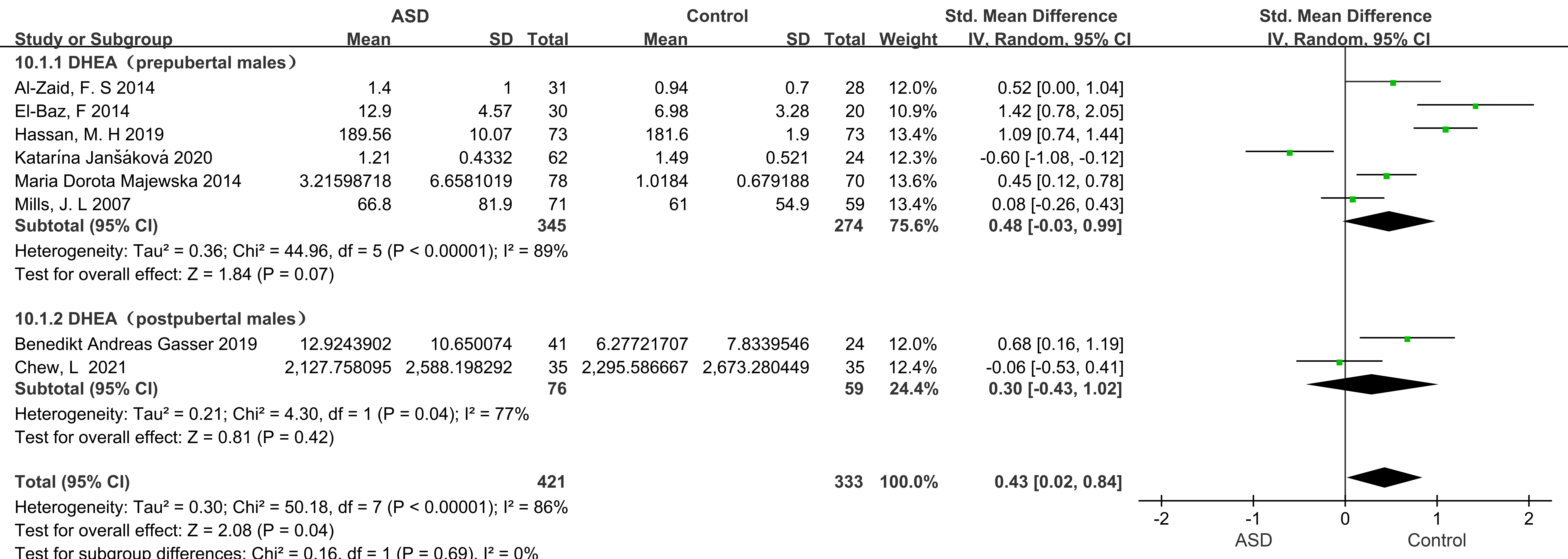

Supplement: Supplementary file 14 [file Image_8.jpeg]

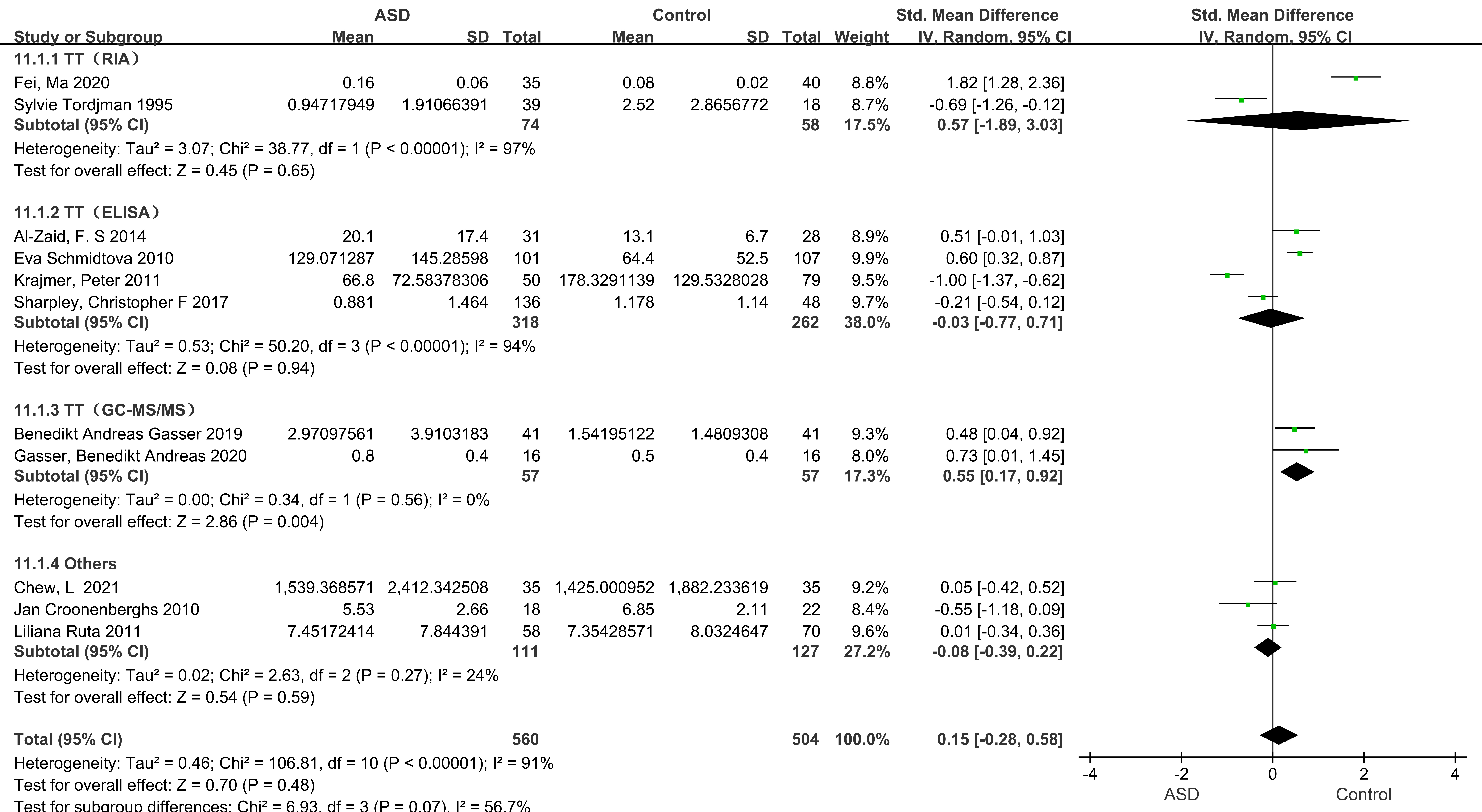

Supplement: Supplementary file 15 [file Image_9.jpeg]

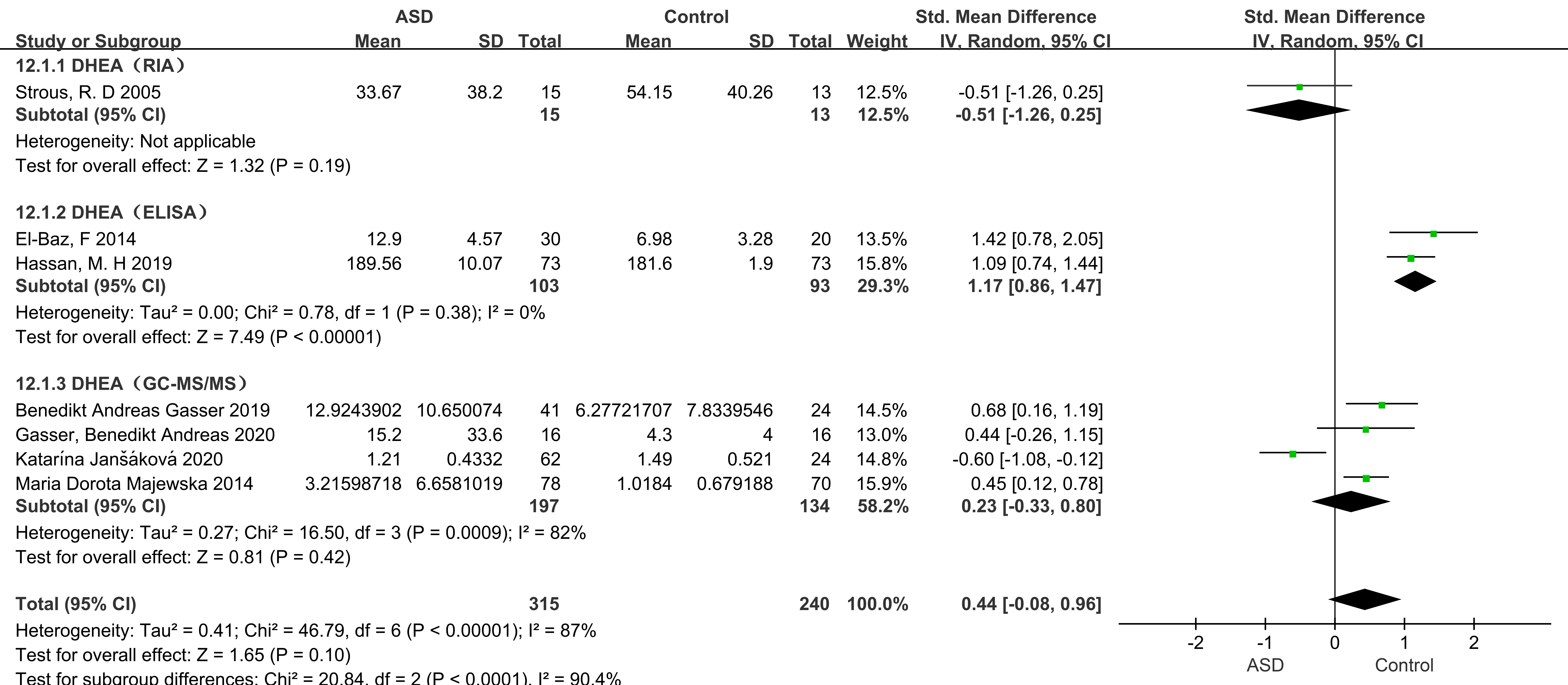

Supplement: Supplementary file 16 [file Image_10.jpeg]

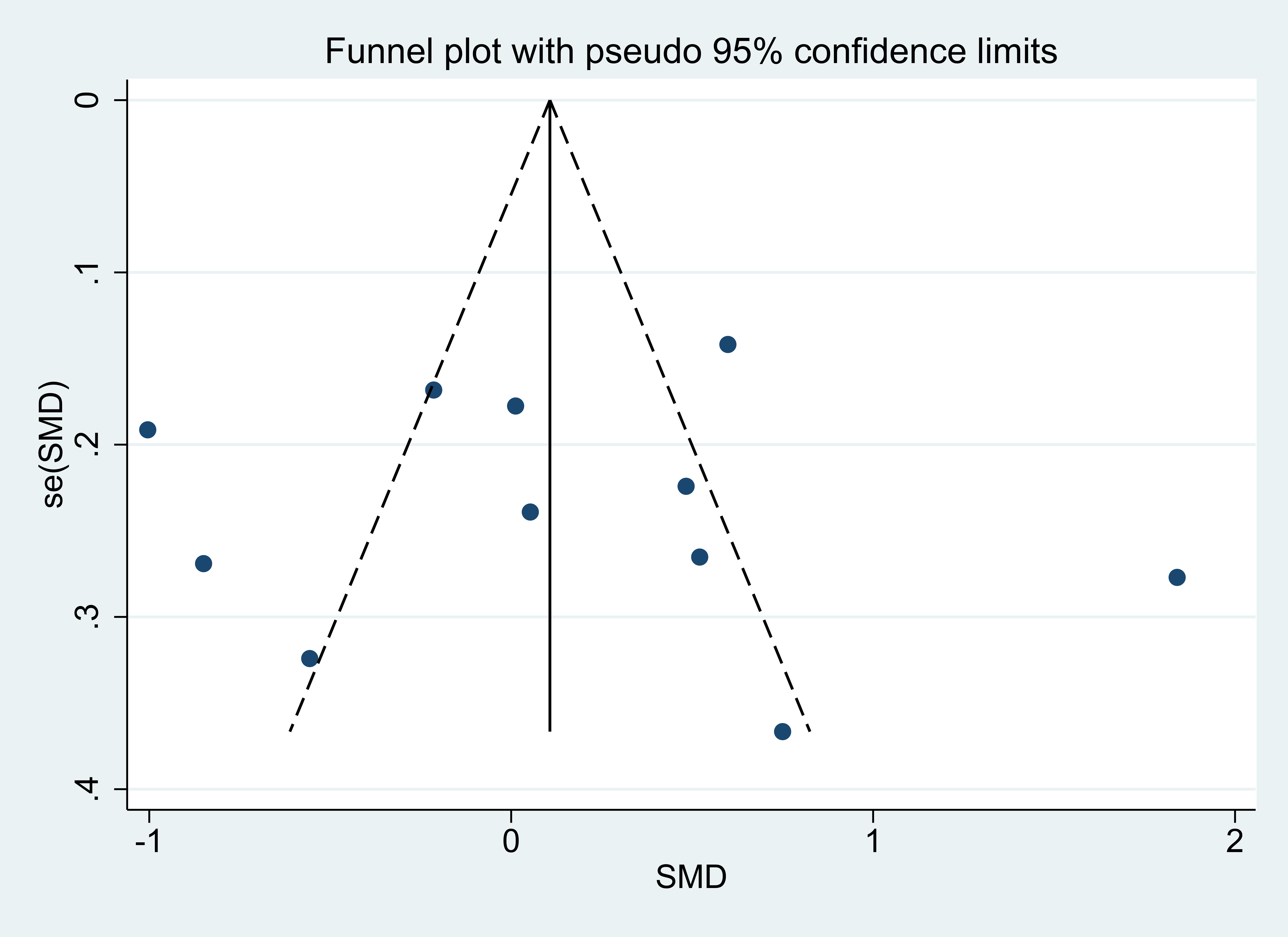

Supplement: Supplementary file 17 [file Image_11.jpg]

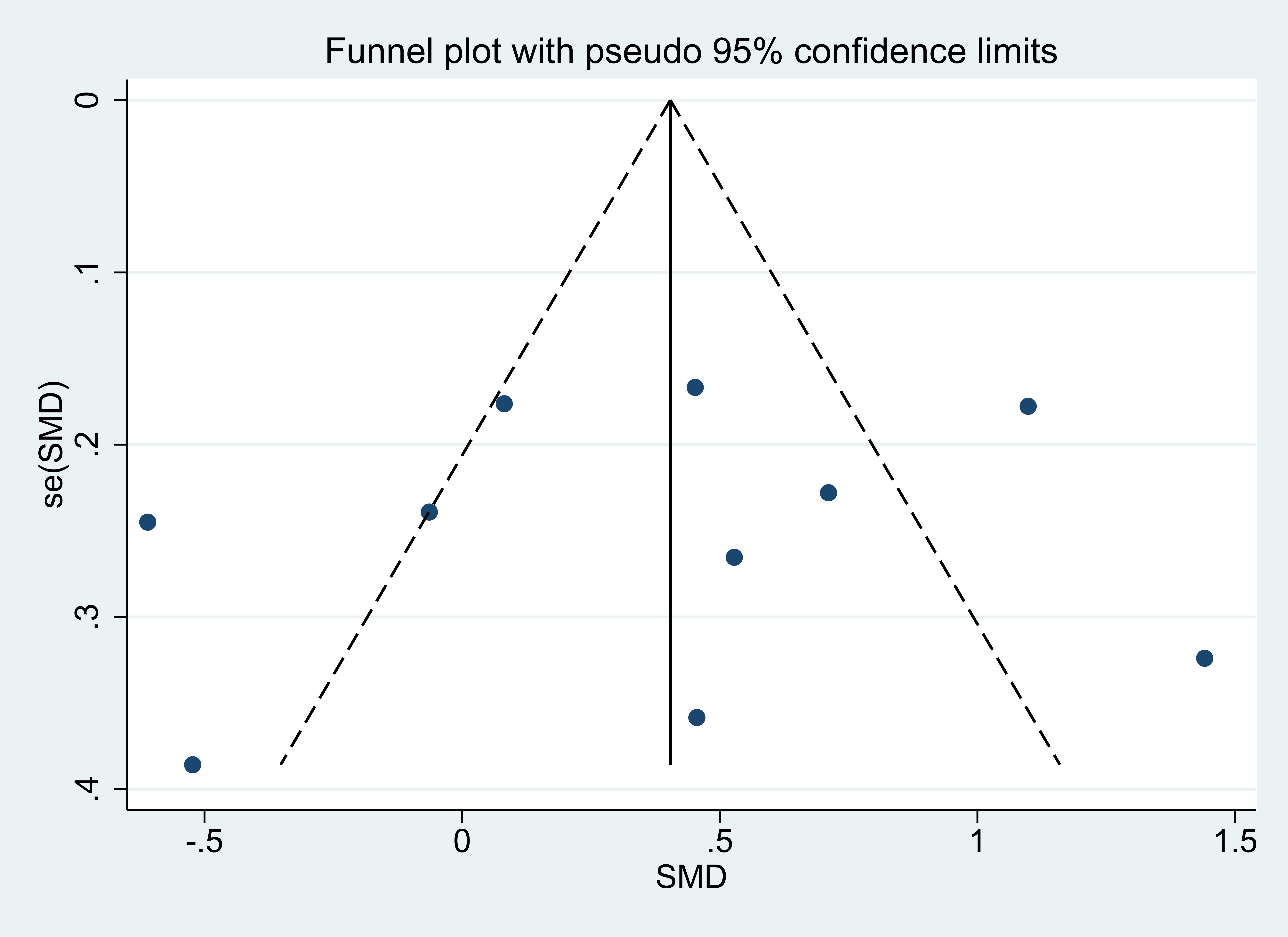

Supplement: Supplementary file 18 [file Image_12.jpg]
